# Effects of iodine intake on gut microbiota and gut metabolites in Hashimoto thyroiditis-diseased humans and mice

**DOI:** 10.1038/s42003-024-05813-6

**Published:** 2024-01-29

**Authors:** Boshen Gong, Fanrui Meng, Xichang Wang, Yutong Han, Wanyu Yang, Chuyuan Wang, Zhongyan Shan

**Affiliations:** Department of Endocrinology and Metabolism, The First Affiliated Hospital of China Medical University, China Medical University, Shenyang, China

**Keywords:** Bacterial host response, Thyroid diseases

## Abstract

Hashimoto thyroiditis (HT) is an organ-specific autoimmune disease linked to iodine intake. Emerging evidence highlights the gut microbiota’s role in HT pathogenesis via the microbiota-gut-thyroid axis. However, the process through which iodine intake modifies the microbiota and triggers HT remains unclear. This study examines how iodine affects gut dysbiosis and HT, recruiting 23 patients with HT and 25 healthy individuals to assess gut microbiota composition and metabolic features. Furthermore, we establish a spontaneously developed thyroiditis mouse model using NOD.H-2h4 mice highlighting the influence of iodine intake on HT progression. The butanoate metabolism significantly differs between these two groups according to the enrichment results, and butyric acid is significantly decreased in patients with HT compared with those in healthy individuals. Gut dysbiosis, driven by excessive iodine intake, disrupts TH17/Treg balance by reducing butyric acid. In summary, iodine intake alters intestinal microbiota composition and metabolic changes influencing the microbiota-gut-thyroid axis.

## Abstract (web summary)

A study on human and mice using 16SrRNA gene sequencing and targeted metabolomic analysis suggests that iodine intake alters intestinal microbiota composition and metabolic changes influencing the microbiota-gut-thyroid axis.

## Introduction

Hashimoto thyroiditis (HT) is an organ-specific autoimmune disease characterized pathologically by infiltrating lymphocytes in the interstitium among thyroid follicles^1^. Most patients with HT ultimately develop hypothyroidism, although they can be euthyroid or hyperthyroid upon diagnosis^2^. HT can result from interactions between environmental factors, genetic factors, imbalance of T cells, and activation of the inflammasome^3,4^. The expression of mRNA and proteins of multiple inflammasomes, including the NLRP3, NLRC4, and AIM2 inflammasomes and their downstream cytokines, was significantly increased in the thyroid tissues of patients with HT^5^. The cellular immune response mediated by T helper lymphocytes TH1 and TH17 can induce HT^6^. Another study showed an increased ratio of Th17/Treg in patients with HT^7^. Short-chain fatty acids (SCFAs) produced by intestinal microbiota, including Bacteroides, can regulate Tregs and Th17 cells in the circulation and extraintestinal tissues^8^.

Iodine is a micronutrient essential for thyroid hormone production, whereas chronic exposure to excess iodine intake leads to HT, partly because highly iodinated thyroglobulin (Tg) is more immunogenic^9^. The primary source of iodine is the diet, which is obtained by consuming foods containing iodine, including salt, fruit, and seafood^10^. Since mandatory universal salt iodization was implemented in China 20 years ago, the Chinese population has been consecutively exposed to iodine nutrition status of excessive iodine intake for 5 years (1996–2001), more than adequate iodine intake for 10 years (2002–2011), and adequate iodine intake for 5 years (2012–2016). This nationally representative cross-sectional study showed that increased iodine intake is significantly associated with elevated serum thyrotropin levels^11^. A cohort study of three regions with different levels of iodine intake in China showed that more than adequate or excessive iodine intake might lead to autoimmune thyroiditis^12^.

Emerging evidence suggests that the gut-thyroid axis plays a significant role in maintaining metabolic and immunological homeostasis in vivo^13^. The microbiota influences thyroid hormone levels by regulating iodine uptake, degradation, and enterohepatic cycling, and altered microbiota composition increases the prevalence of HT^14^. A damaged intestinal barrier increases intestinal permeability, allowing antigens to enter the circulatory system and activate the immune system or cross-react with extraintestinal tissues^15,16^. Several studies have investigated alterations in the gut microbiota between patients with HT and healthy individuals^17^. The richness and diversity of the microbiota were significantly lower in patients with HT than in controls^18^. Zhao et al. revealed that the abundance levels of *Blautia*, *Roseburia*, *Romboutsia*, and *Dorea* increased in patients with HT, whereas those of *Fecalibacterium* and *Bacteroides* decreased^19^. However, the mechanism through which iodine intake alters the microbiota and causes HT remains unclear. To this end, this study aimed to examine how iodine affects gut dysbiosis and causes HT.

## Results

### Significant differences in gut microbiota between patients with HT and healthy individuals

**Fig. 1:**
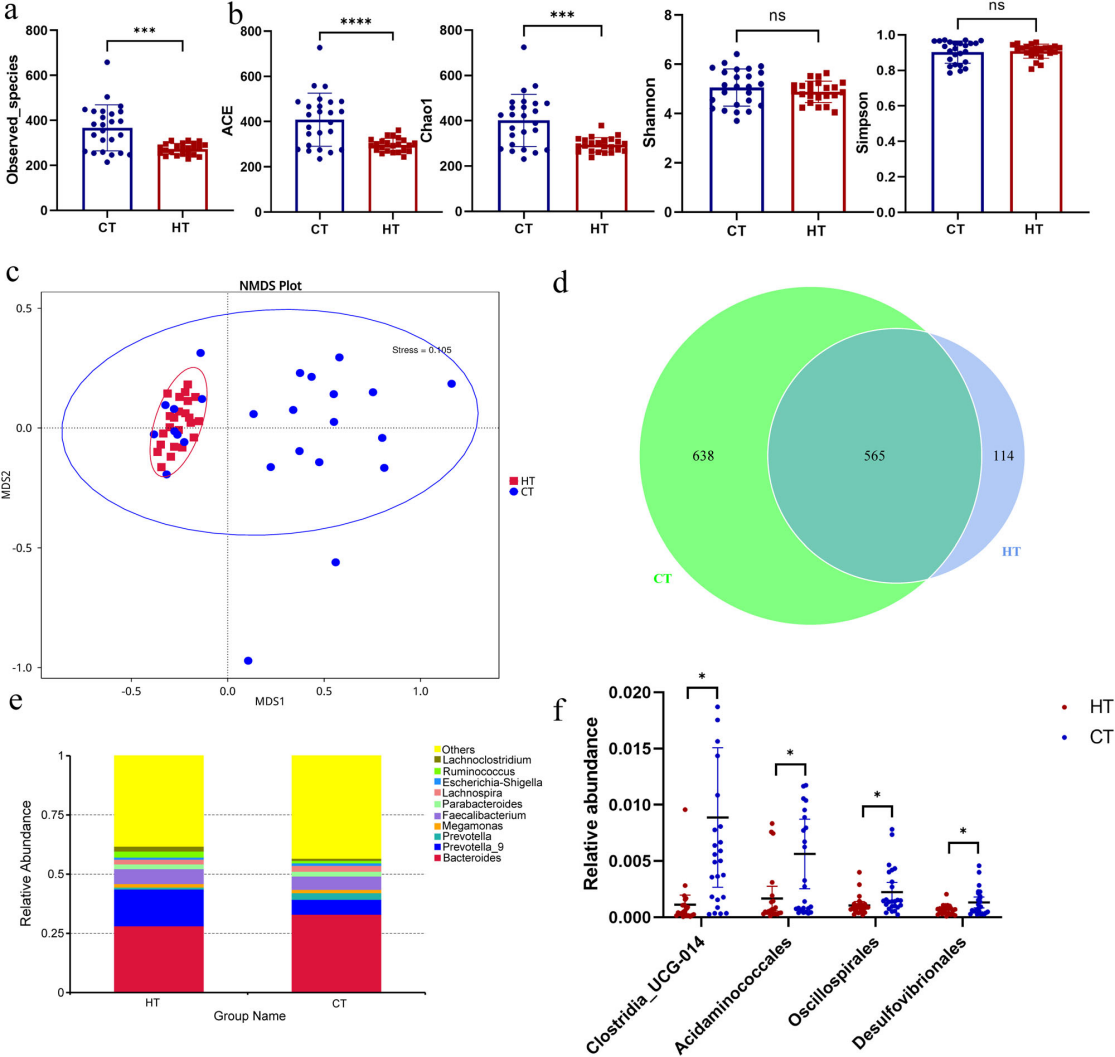
Significant changes in the gut microbiota between patients with HT and healthy controls. **a** Observed species between healthy control (CT) and patients with HT (HT). **b** The α diversity indices (Chao 1, ACE, Shannon, and Simpson indexes) of the microbiota in healthy control (CT) and patients with HT (HT). **c** Non-metric multidimensional scaling (NMDS) plot based on Bray–Curtis distance matrix of control and HT groups. **d** The Venn diagram illustrates the overlap of OTUs in gut microbiota among the two groups. Overlapping parts in the figure indicate common species. **e** Genus level comparison of fecal microbiota between the HT and healthy groups. **f** Comparison of the bacteria abundance at the order level in patients with HT and healthy controls using *t* test. *N* = 23 and 25 for HT and healthy control groups respectively.

**Fig. 2:**
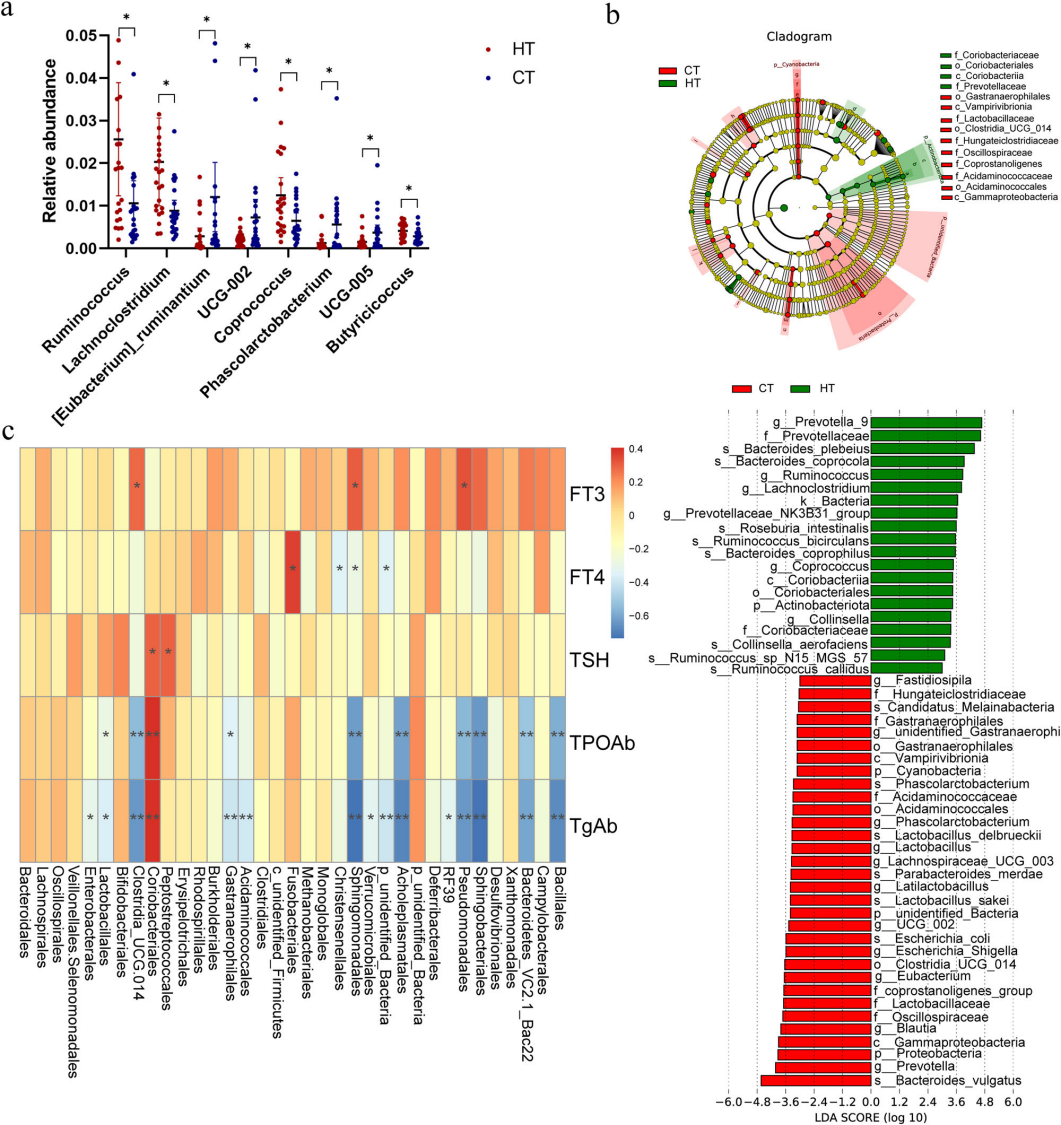
Alterations of the gut microbiota in patients with HT compared to healthy controls. **a** Comparison of the bacteria abundance at the genus level in patients with HT and healthy controls by *t* test. **b** Cladogram generated using LEfSe analysis. Red: increased abundance in the control group. Green: increased abundance in the HT group. LDA scores of the microbiota in HT and control groups at different taxonomic levels using LEfSe analysis. LDA score >3 or <3 represents bacteria taxa that are significantly enriched in the HT group (green) or control group (red) (*P* < 0.05). **c** Heatmap of correlations between thyroid clinical indicators of HT and the abundances of gut microbiota.

To clarify the role of the microbiota in the pathogenesis of HT, we used 16Sr RNA gene sequencing to analyze fecal samples from patients with HT and healthy controls. The good’s coverage index of the sample was 0.99, which was used to represent the sequencing depth. A rarefaction curve was utilized to demonstrate the adequacy of the sequencing data (Fig. S1a), and rank-abundance curves, based on OTU level, indicated that the species distribution was both even and rich (Fig. S1b). No significant difference was observed in the sequencing depth between the HT and healthy groups (*P* > 0.05). Alpha diversity, including community richness (such as, number of observed features) and diversity (Shannon and Simpson indices), showed an overall increasing trend and was applied to analyze the complexity of species diversity in each sample. These indices were calculated for our samples using QIIME (version 1.9.1) based on rarefied OTU counts. The number of observed bacterial species was significantly decreased in patients with HT (Fig. 1a), and Abundance Coverage-based Estimator (ACE), and Chao 1 indices were also decreased compared to healthy controls (Fig. 1b). Conversely, no significant differences were observed in Shannon and Simpson diversity indices between the two groups. The microbiota structural changes were analyzed using non-metric multidimensional scaling, and there was a noticeable separation between the samples of patients with HT and healthy controls, indicating that the microbiota composition significantly differed from each other (Fig. 1c). Approximately 114 and 638 of the OTUs were identified in the samples from patients with HT and healthy controls, respectively (Fig. 1d). The composition and structure of the gut microbiota in patients with HT differed significantly from those in healthy controls at the genus level (Fig. 1e). Random forest analysis showed the top 20 bacteria that distinguished patients with HT from the healthy group, with an area under the curve value of 99.17% (Fig. S1c). The top 25 relative abundance of intestinal bacteria at the phylum level are shown in Fig. S1d. We found that the microbiota composition between the patients with HT and healthy controls was significantly different. At the order level, the relative abundance of *Clostridia_UCG-014*, *Acidaminococcales*, *Oscillospirales*, and *Desulfovibrionales* significantly decreased in patients with HT compared with that in healthy controls (Fig. 1f), and the different microbiota at the genus level are shown in Fig. 2a. LEfSe analysis demonstrated that the phylum *Actinobacteriota (p_ Actinobacteriota)* and family *Prevotellaceae* (f_ *Prevotellaceae*) were enriched in patients with HT. However, the phylum *Proteobacteria (p_ Proteobacteria)*, *Cyanobacteria (p_ Cyanobacteria)*, order *Clostridia_UCG_014 (o_ Clostridia_UCG_014)*, class *Vampirivibrionia (c_Vampirivibrionia)*, and family *Lactobacillaceae (f_ Lactobacillaceae)*, *Acidaminococcaceae (f_ Acidaminococcaceae)* were enriched in the healthy control group (Fig. 2b). Spearman’s correlation analysis was used to explore the potential correlation between different bacterial abundances and clinical thyroid-related indicators (Fig. 2c). The analysis of gut microbiota in patients with HT between the two sexes is illustrated in Fig. S2a–d.

### Gut metabolites were markedly changed in patients with HT

**Fig. 3:**
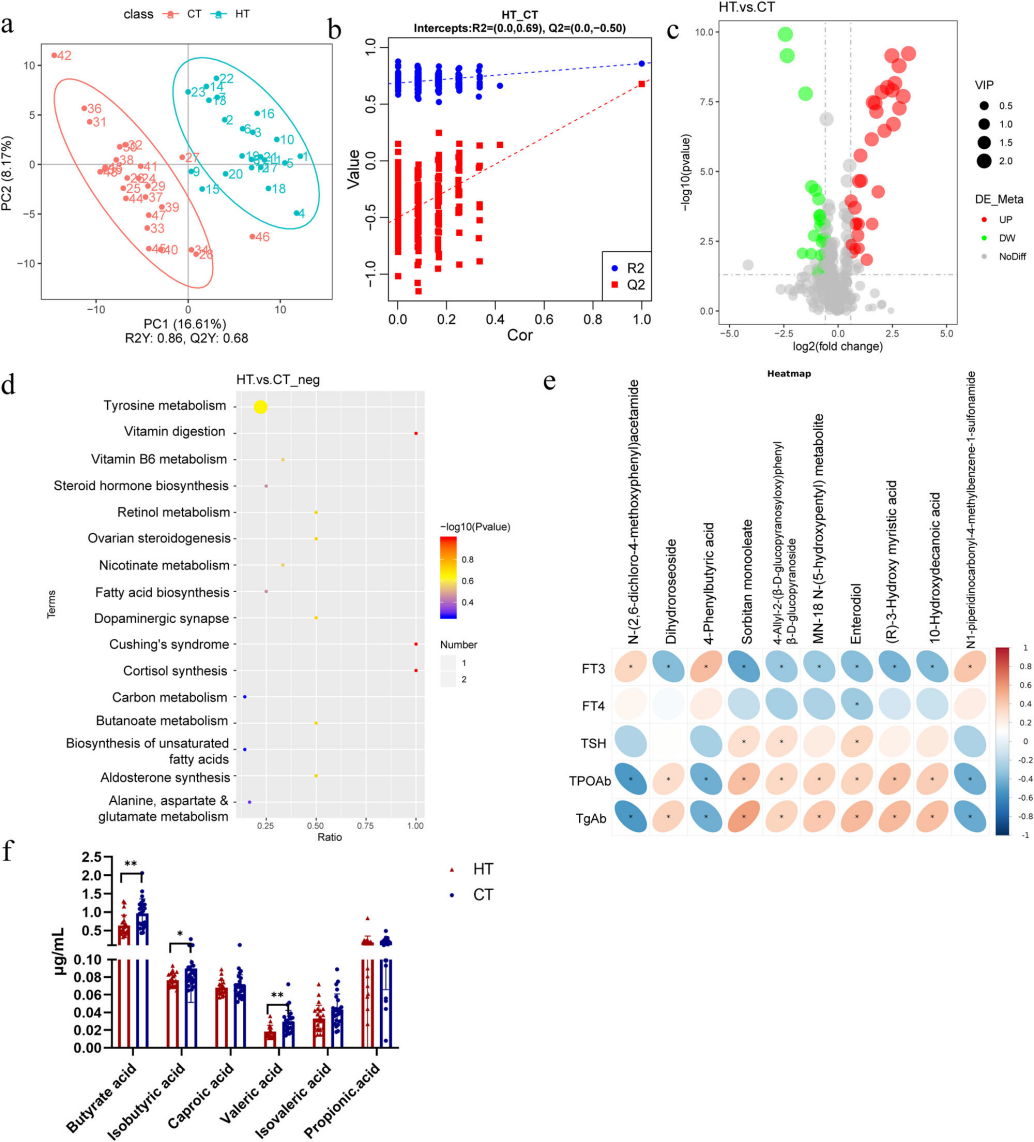
Significant changes in the metabolism between HT and healthy control groups. **a** PLS-DA between the control and HT groups (negative mode). **b** Validation of the PLS-DA model (negative mode). **c** Volcano map of the metabolites screened between the HT and control groups and was built based on log2(fold change) and -log10(p-value) (negative mode). **d** The top 16 KEGG pathways related to differentially expressed metabolites between the HT and control groups. **e** Heatmap of correlations between the five clinical indicators of HT and the significantly changed metabolites in patients with HT. The color bar with numbers indicates the correlation coefficients. **f** The concentrations of SCFAs in the serum of HT and control groups were determined by GC-MS.

Gut metabolism is an important bridge for gut microbiota to balance the host immune system. Kyoto Encyclopedia of Genes and Genomes (KEGG) and untargeted metabolomic analyses were used to investigate the related metabolic pathways in patients with HT and healthy controls. We analyzed the metabolites in positive ion mode (POS) and negative ion mode (NEG). The POS and NEG modes are commonly used to analyze the metabolic profiles of samples using a traditional UHPLC-QTOF/MS approach. Our study revealed that 54 differentially expressed metabolites (31 upregulated and 23 downregulated) were significantly altered in the NEG mode, and 108 differentially expressed metabolites (74 upregulated and 34 downregulated) were significantly altered in the POS mode. A PLS-DA score plot revealed a visible separation of serum metabolites between the control and HT groups (Fig. 3a). Permutation tests confirmed that the PLS-DA model did not overfit the data, revealing significant metabolic variations between groups (Fig. 3b). In the volcano plot, variables significantly contributing to the clustering and discrimination were identified according to a variable importance in the projection threshold of ≥1.0, with a *P* < 0.05 (Fig. 3c). The heatmap of the differentially expressed metabolites indicating that the metabolic profiles of patients with HT markedly changed compared with the control group (Fig. S2e). The results of the positive mode are shown in Fig. S3a–d. KEGG pathway analysis was applied to determine biochemical metabolic pathways and signal transduction pathways related to the differentially expressed metabolites between the HT and control groups. According to the enrichment results, butanoate metabolism in the HT group was altered compared to that in the control group (Fig. 3d). Changes in the tyrosine and vitamin B6 metabolism pathways were also observed. *Enterobacterales* and *Monoglobales* were positively associated with 4-Phenylbutyric acid. The abundance of *Clostridia_UCG-*014 significantly decreased in patients with HT and was positively associated with N-(2,6-dichloro-4-methoxyphenyl) acetamide, which was negatively associated with TPOAb and TgAb (Fig. S3e). Many remarkably altered metabolites were significantly correlated with clinical indicators of patients with HT. N-(2,6-dichloro-4-methoxyphenyl) and 4-Phenylbutyric acid were strongly negatively correlated with TPOAb and TgAb, whereas Dihydroroseoside and Sorbitan monooleate were strongly positively correlated with TPOAb and TgAb (Fig. 3e). We further detected the SCFA using GC-MS, and the results demonstrated a significant decrease in butyric acid levels in HT patients compared to healthy individuals (Fig. 3f). Furthermore, in iodine-induced HT mice, there was a significant increase in the serum level of LPS (Fig. S3f).

### Iodine intake changed the composition of gut microbiota and metabolites in the HT mouse model

**Fig. 4:**
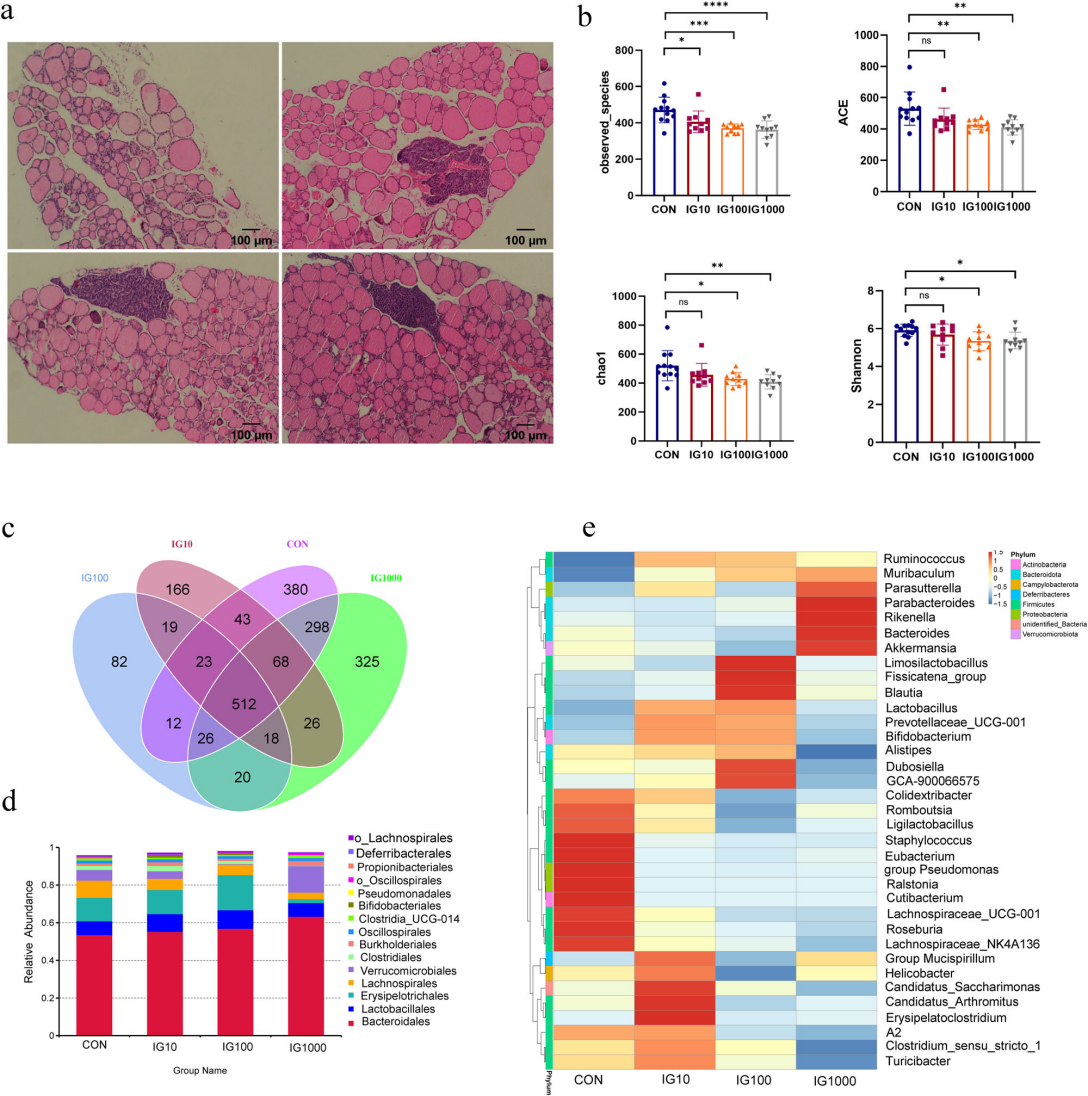
Different iodine intake changed the composition of gut microbiota in HT mice. **a** Thyroid inflammation was determined according to the lymphocyte infiltration area using HE staining of the mice’s thyroid gland. Scale bar: 100 μm. **b** The α diversity indices (Chao 1, ACE, Shannon, and Simpson indexes) of the microbiota among different iodine intake groups. **c** The Venn diagram illustrates the overlap of OTUs in gut microbiota among different iodine intake groups. Overlapping parts in the figure indicate common species. **d** Order level comparison of fecal microbiota among these four groups. **e** Heatmap based on the abundance of the first 35 genera among these four groups. *N* = 12, 10, 10, 10 for CON, IG10, IG100, and IG1000 groups respectively.

To explore the mechanisms through which iodine influences the gut microbiota composition, we used the HT model in humans, NOD.H-2h4 mice, which express I-A(k) on a NOD background, spontaneously developing autoimmune thyroiditis. All the male and female mice used in this study were aged 5 to 6 weeks. We added different amounts of iodine 5 mg/L (IG10), 50 mg/L (IG100), and 500 mg/L (IG1000) to the drinking water of NOD.H2-h4 mice for 10 weeks, respectively. The flattening of rarefaction curves for each group, along with the rank-abundance curves based on OTU level, indicated adequate sequencing depth (Fig. S4a, b). Thyroid inflammation was determined based on the lymphocyte infiltration area using HE staining (Fig. 4a). Compared with the control groups, the scoring of lymphocyte infiltration of the thyroid gland and the serum TgAb concentration were significantly increased (Fig. S4c). Notably, the observed species and α diversity (ACE, Chao1, and Shannon indices) decreased with increasing iodine intake (Fig. 4b). The Venn diagram shows the common and unique numbers of OTU in each group (Fig. 4c). The PCoA plot based on binary Jaccard distance revealed that the microbiota composition of different iodine intake groups significantly differed from that of the control group (Fig. S4d). LEfSe analysis revealed that the genera *Roseburia* and *Staphylococcus* were enriched in the control group. However, the genus *Turibacter* was enriched in the IG10 group, the family *Erysipelotrichaceae* was enriched in the IG100 group, and the genera *Akkermansia* and *Bacteroides* were enriched in the IG1000 group (Fig. S4e, f). The composition and structure of the gut microbiota among the four groups were significantly different at the order level (Fig. 4d). A heatmap generated based on the relative abundance of the first 35 genera showed that at the genus level, the relative abundance of *Roseburia* and *Cutibacterium* were the predominant phyla in the control group, *Erysipelatoclostridium, Blautia*, and *Akkermansia* in the IG10, IG100, and IG1000 groups, respectively (Fig. 4e). Analysis of gut microbiota in the HT mouse model across different genders is depicted in Fig. S5a–d.

### Butyrate rebalanced the composition of HT mice and enhanced the gut barrier integrity

**Fig. 5:**
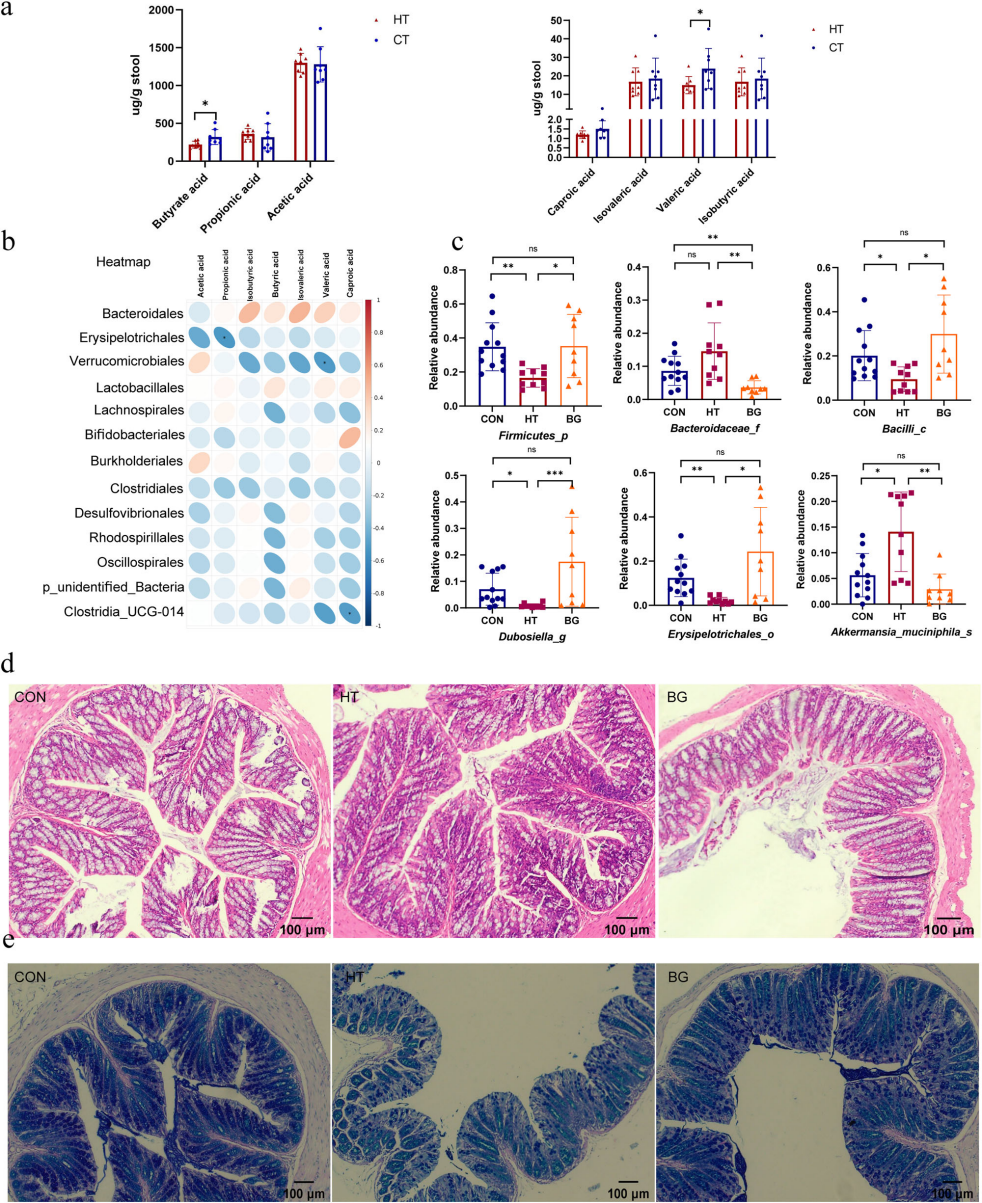
Butyric acid altered the composition of gut microbiota of HT mice, and enhanced gut barrier integrity. **a** The concentration of butyric, propionic, acetic, caproic, isovaleric, valeric, and isobutyric acids in fecal samples of the HT and control groups were determined by GC-MS. (*N* = 8 in each group). **b** Heatmap of correlations between the significantly changed gut microbiota and seven SCFA metabolites in HT mice at the family level. The color bar with numbers indicates the correlation coefficients. **c** Relative abundances of the following six significantly altered microbiota among the three groups: *Firmicutes, Bacteroidaceae, Bacilli, Dubosiella, Erysipelotrichales*, and *Akkermansia_muciniphila*. **d** Histological changes were observed using HE staining, and goblet cells are round cells that appear clear on HE staining and are typically flanked by the purplish absorptive-type cells. Scale bar: 100 μm. **e** AB/PAS staining on intestine tissue sections was performed to evaluate the histopathology of the intestines. *N* = 7 for CON group, 7 for HT group, and 8 for BG group. The Kruskal-Wallis test was used to detect significant changes for *Bacilli, Dubosiella, Erysipelotrichales*, and *Akkermansia_muciniphila*. The Welch’s ANOVA test was used for *Firmicutes*,and *Bacteroidaceae*. ^*^P < 0.05, ^**^P < 0.01, ^,^ and ^***^P < 0.001, ns, no significance.

SCFAs in the intestine were detected using GC-MS, and the results showed that butyrate and valeric acids were significantly decreased in iodine-induced HT mice than that in control group (Fig. 5a). Butyric acid was significantly positively correlated with SCFA-producing bacteria, including *Muribaculaceae* and *Prevotellaceae*, whereas valeric acid was positively correlated with *Muribaculaceae, Prevotellaceae, Tannerellaceae* and negatively correlated with *Akkermansiaceae* (Fig. 5b). The levels of other SCFAs, including caproic, isovaleric, and isobutyric acids, decreased without statistical significance in the HT group. To clarify butyrate’s role in microbiota composition in HT mice, we added sodium butyrate to the drinking water of NOD.H-2h4 mice. The composition and structure of the gut microbiota in the healthy, HT, and butyrate groups differed significantly at the phylum level. The relative abundance of *Firmicutes* increased compared to that in the HT and control groups after feeding sodium butyrate in drinking water for 10 weeks (Fig. S6a). The PCoA plot based on the binary Jaccard distance revealed that microbiota composition among these three groups differed significantly (Fig. S6b). LEfSe analysis revealed that bacteria, including *Firmicutes* and *Bifidobacteriaceae*, were enriched in the butyrate group (Fig. S6c, d). The observed species and α diversity (ACE, Chao1, and Shannon indexes) decreased in the HT group compared with the control group. However, the α diversity in the butyrate group increased compared to that in the HT group without statistical significance (Fig. S6e). Significant differences among the three groups were detected for *Firmicutes* (*P* = 0.0108), *Bacteroidaceae* (*P* = 0.0030), *Bacilli* (*P* = 0.0108), *Dubosiella* (*P* = 0.0005), *Erysipelotrichales* (*P* = 0.0112), and *Akkermansia_muciniphila* (*P* = 0.0007). We further selected six genera and used boxplots to display detailed information (Fig. 5c). *Ligilactobacillus* correlated with butyric, valeric, and caproic acids (Fig. S7a). The relative abundances of the top 35 microbial genera within each group are illustrated in Fig. S7b. The butyrate group had specific network of gut microbiota at the genus level, comprising mainly *Firmicutes* (butyrate-producing bacteria) (Fig. S7c). Histological changes were observed using HE staining, and goblet cells were observed using AB/PAS staining (Fig. 5d, e). The statistical results of goblet cell density by AB/PAS were shown in Fig. S7d. AB/PAS staining of intestinal tissue sections was performed based on the histopathology of the intestines. Goblet cells are round cells that appear clear HE and stain indigo blue in AB/PAS staining, respectively. Butyrate acid was more prominent in maintaining colonic goblet cell function and enhancing gut barrier integrity.

### Butyrate modulates Th17/Treg balance through FFAR2 and FFAR3

**Fig. 6:**
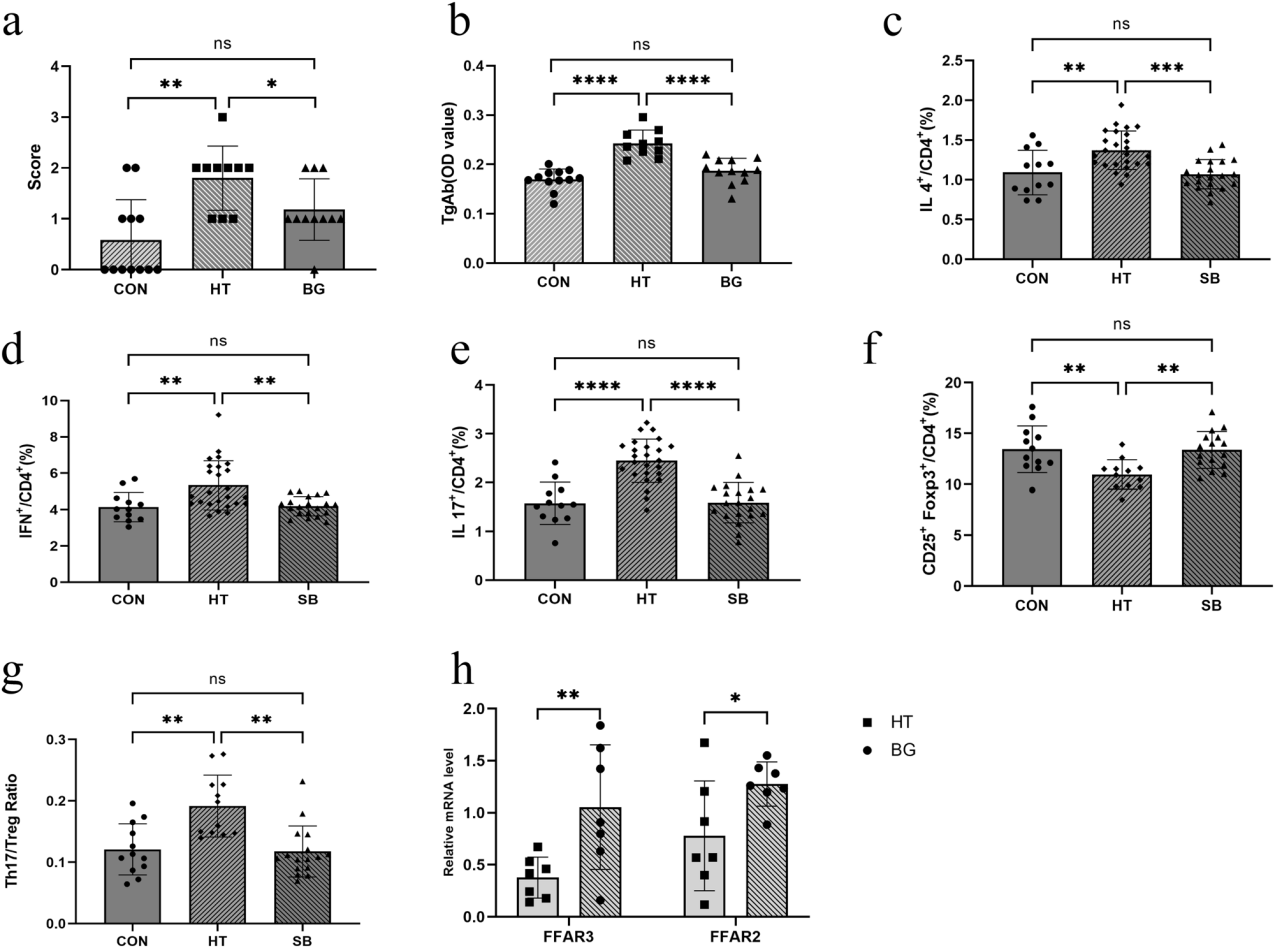
Butyrate acid modulates the Th17/Treg imbalance in the spleen of mice assessed using flow cytometry. **a** The scoring of lymphocyte infiltration of thyroid gland serum. **b** TgAb concentrations were determined using ELISA. **c** The percentage of CD4 + IL4 + T cells (Th2) among these three groups analyzed using flow cytometry. **d** The percentage of CD4 + IFN^+^cells (Th1) among these three groups analyzed using flow cytometry. **e** The percentage of CD4 + IL17+ cells (Th17) among these three groups analyzed using flow cytometry. (*N* = 12, 25, 20 for CON, HT, and BG group respectively). **f** The percentage of CD4 + CD25 + FOXP3+ cells (Treg) among these three groups analyzed using flow cytometry. **g** The ratio of TH17/Treg (CD4 + IL17 + / CD4 + CD25 + FOXP3+ cells) significantly decreased in the butyrate group compared with the HT group. **h** The mRNA expression of FFAR3 and FFAR2 in mice colon tissue. *N* = 7 in each group. The Kruskal-Wallis test was used to detect significant changes for (**a**, **d**), and One-way ANOVA was used for (**b**), (**c**), (**e**), (**f**). The Welch’s ANOVA test was used for (**g**). ^*^P < 0.05, ^**^P < 0.01, ^***^P < 0.001, and ^****^P < 0.0001, ns, no significance.

In our study, we found that the lymphocyte infiltration area and scoring of the thyroid were significantly decreased in the butyrate group compared with the HT group (Figs. 6a and S7e). Consistent with the inflammatory score of the thyroid, serum TgAb concentration in the butyrate group was significantly lower than that in the HT group (Fig. 6b). Flow cytometry analysis was used to investigate the effect of butyrate on immune balance. Compared to the healthy group, the percentage of Th1 and Th2 cells increased in the HT group. The percentage of Tregs (CD4 + CD25 + FOXP3 + ) significantly decreased, whereas the percentage of Th17 (CD4 + IL17 + ) cells was higher in the HT group than in the control group. The Th17/Treg ratio increased compared with that in the control group, whereas the ratio significantly decreased in the sodium butyrate group (Fig. 6c–g). Butyrate acid may signal through cell surface G-protein-coupled receptors, including *FFAR2* and *FFAR3*, to activate signaling cascades that control immune functions. In our study, we found that the expression of *FFAR2* and *FFAR3* mRNA in the butyrate group was markedly upregulated compared to that in the HT group (Fig. 6h).

## Discussion

To date, no study has indicated a direct association between the gut microbiota, which is altered by iodine intake, and the pathogenesis of HT. Therefore, to bridge this gap, we used the 16S rRNA sequencing technique and revealed that iodine intake altered the composition and metabolism of the gut microbiota, which is an important pathogenic factor in the development and immune abnormalities of HT. The composition and metabolism of the intestinal microbiota in patients with HT, particularly butanoate metabolism, were significantly different from those in healthy controls. Our study found that the relative abundance of *Clostridium*, a reported potential butyrate producer, was significantly decreased in patients with HT and the HT mouse model compared with the control group^20^. Furthermore, we established an iodine-induced HT mouse model associated with gut microbiota dysbiosis with NOD.H-2h4 mice by feeding them water with different NaI concentrations for 10 weeks. Iodine intake can alter the composition and metabolism of the gut microbiota, for example, by decreasing the abundance of butyrate-producing bacteria. After feeding 120 mM sodium butyrate into drinking water, sodium butyrate significantly ameliorated intestinal epithelial barrier dysfunction and reshaped the composition of the gut microbiota by elevating the abundance of beneficial bacteria in the HT mouse model. Sodium butyrate modulated the balance between Th17 and Treg cells in vivo in the HT mice through cell surface *FFAR2* and *FFAR3*.

HT is the most common autoimmune disease worldwide, characterized by chronic inflammation and circulating autoantibodies against thyroid peroxidase and Tg^1^. Emerging studies have provided a preliminary understanding of the thyroid-gut axis, indicating that the intestinal microbiota and its metabolites may act directly or indirectly on the thyroid by influencing intestinal microelement uptake and immune regulation^21^. Alterations in the gut microbiota and increased gut permeability are involved in the development of many autoimmune and chronic inflammatory diseases^22^. A cohort study conducted in China showed that more than adequate or excessive iodine intake might lead to HT; however, the mechanism by which iodine intake influences the thyroid-gut axis has not yet been elucidated. A descriptive study reported that intestinal dysbiosis and increased intestinal permeability favor HT development but did not explore the association between iodine intake and the microbiota of HT^17^. *Clostridia_UCG-014* is the key bacteria closely correlated with the development of rheumatoid arthritis and was reported to be significantly decreased compared with the healthy group^23^. In our study, *Clostridia_UCG-014* was negatively correlated with TPOAb and TgAb, and significantly decreased in the patients with HT, compared with the healthy controls. Besides, the relative abundance of *Desulfovibrionales was* significantly decreased in patients with HT compared with the healthy controls. Notably, *Desulfovibrionales* was enriched in the butyrate group in mice.

Several studies have explored the alterations in the microbiota composition of HT patients, while no study has explored the sex differences in microbiota among HT patients^24^. Given the significant sex-dependent differences in the prevalence and incidence of HT, we analyzed the composition of gut microbiota in HT patients of different genders^25^. The composition and structure of the gut microbiota in males and females with HT were similar at the order level. Approximately 126 and 102 OTUs were identified in male and female patients with HT, respectively. LEfSe analysis demonstrated that *Desulfovibrionia* was enriched in the male HT group, and *Enterobacteriaceae* in the female group. However, no significant difference in α diversity was found between sexes in patients with HT. In the HT mouse model, approximately 176 and 349 OTUs were identified in male and female mice with HT, respectively. The composition and structure of the gut microbiota between male and female HT mice differs at the phylum level. LEfSe analysis revealed that the genera *Dubosiella* and *Bifidobacterium* were enriched in male HT mice, while the families *Bacteroidaceae* and *Ruminococcaceae* were enriched in female HT mice.

Iodine forms part of the thyroid hormone structure and is essential for cell metabolism and differentiation^26^. Regarding nutritional factors, evidence suggests that high iodine intake induces HT, partly because high Tg levels are more immunogenic^27^. At the population level, excess iodine intake may arise from consuming over-iodized salt, drinking water, iodine-containing dietary supplements, excess seaweed, or a combination of these sources^28,29^. A cross-sectional study found that participants with more than adequate and excessive intake had a higher incidence of HT than those with mildly deficient iodine intake^12^. However, the mechanism by which iodine intake promotes HT progression has not yet been fully elucidated. A previous study found that iodine treatment increased thyroid hormone concentrations, modulated the gut microbiota with an increased abundance of pathogenic bacteria, and decreased the proportion of beneficial bacteria in an obese mouse model^30^. Microbiota can influence thyroid hormone levels by regulating iodine uptake, degradation, and enterohepatic cycling^14^.

SCFAs are the key products of the microbiota, including butyrate, acetate, and propionate acids^31^. Butyrate acid is known to possess anti-inflammatory properties and inhibits the expression of pro-inflammatory cytokines and chemokines in autoimmune diseases^32^. Gut microbiota also plays a role in synthesizing vitamins B and K and the metabolism of bile acids, and other sterols^33^. Studies reported that several metabolisms, including vitamin D and tyrosine, significantly differ between patients with HT and healthy controls^34,35^. In our study, we found that butanoate metabolism and butyric acid were significantly altered in patients with HT compared with healthy controls. Furthermore, butyric acid levels were significantly lower in HT mice than in the control group. Butyric acid acts as a key mediator of host-microbe crosstalk through specific receptors (GPR43, GPR41, and GPR109a)^31^. Butyrate inhibits histone deacetylase and promotes a unique chromatin landscape in T-cells^36^. In addition to directly regulating the inflammatory and immune systems, butyrate can enhance gut barrier integrity. Here, we found that beneficial bacteria, including *Firmicutes*, *Bacilli*, and *Bifidobacterium*, were enriched in the sodium butyrate group. We assumed that the reduction in butyrate-producing bacteria led to decreased SCFAs, which might be involved in impairing gut barrier integrity.

An imbalance in the differentiation of effector Th1, Th2, Th17, and Treg lymphocytes is associated with the pathological processes of HT^37^. A study using single-cell RNA sequencing found that immune-dysregulated cells interact and activate relevant immune pathways and further aggravate the immune response in patients^38^. A decrease in Treg cells and an increase in Th17 cells in the peripheral blood of patients with HT have been previously reported^39,40^. Evidence shows that an imbalance in the Th17/Treg ratio is a prognostic factor for thyroid gland damage^41^. Butyric acid can enhance Tregs over inflammatory Th cells, including Th17 cells^42^. In our study, we found that sodium butyrate treatment can regulate the Th17/Treg imbalance and alleviate lymphocyte infiltration in HT mice. The percentages of Th1 and Th2 cells were lower in the sodium butyrate group than in the HT group.

**Fig. 7:**
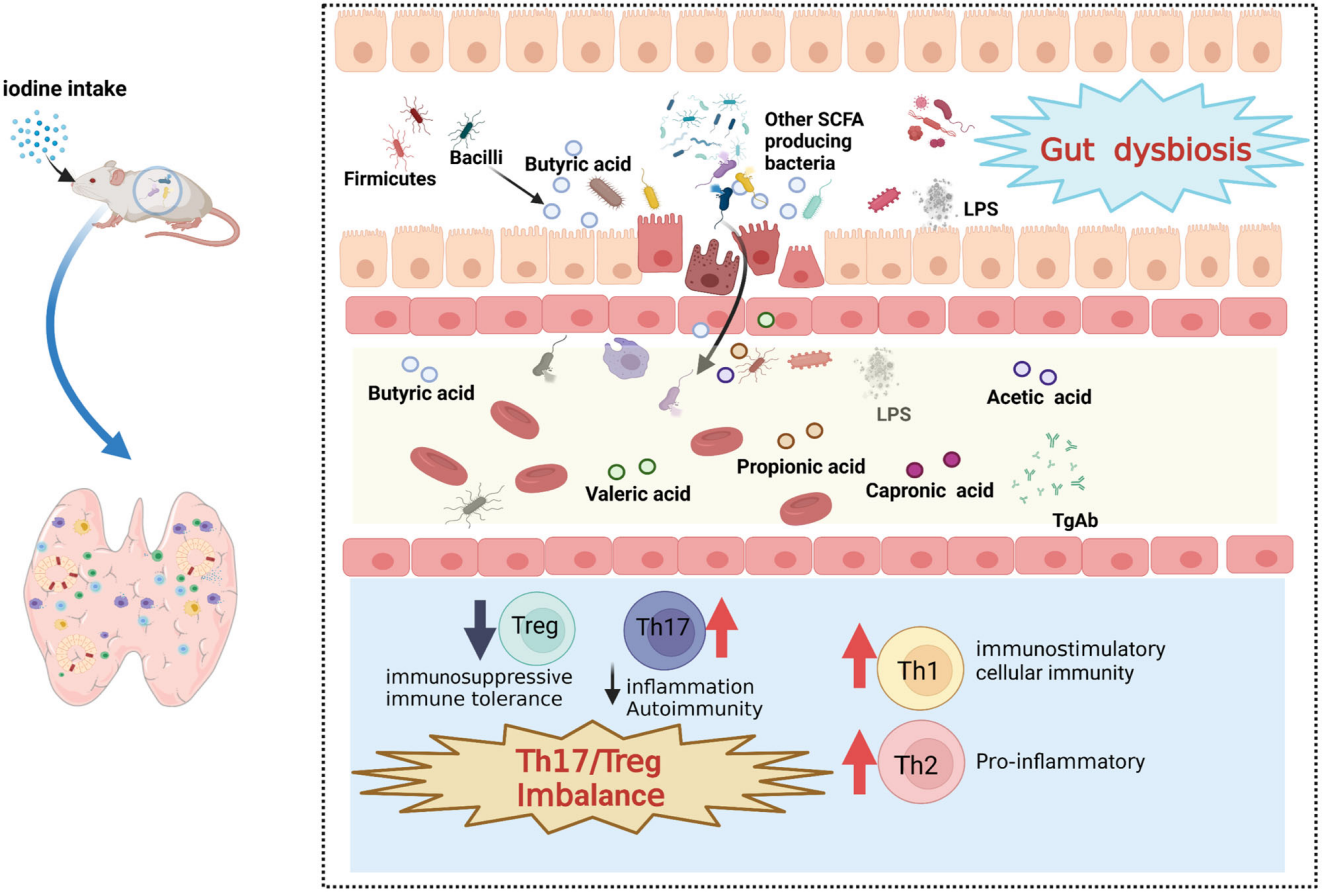
Schematic representation of the mechanism of how iodine intake altered the composition of gut microbiota and metabolites to accelerate the progress of HT. Thyroid-gut axis plays an important role in the development of HT, indicating that the intestinal microbiota and its metabolites may act directly or indirectly on the thyroid by influencing intestinal microelement uptake and immune regulation. Excess iodine intake increases intestinal permeability, and leads to gut dysbiosis, disrupting TH17/Treg balance by reducing butyric acid. The blue downward arrows indicate downregulation/decrease. The red upward arrows indicate upregulation/increase. Abbreviation: HT, Hashimoto thyroiditis; LPS: Lipopolysaccharide; TgAb: thyroglobulin antibody.

Our study revealed an interactive association between iodine intake and gut microbiota in HT immune dysfunction (Fig. 7). The composition and metabolism of the intestinal microbiota in patients with HT were significantly different from those in healthy controls, particularly the butyrate-producing bacteria and butanoate metabolism. NOD.H-2h4 mice were used to explore the influence of iodine intake on microbiota composition and intestinal metabolism. We provide valuable insights into the microbiota-gut-thyroid axis in patients with HT and evidence for the national normalization for iodine intake standards.

## Methods

### Ethics statement

The study was approved by the Medical Ethics Committee of China Medical University (No. 2022-247). All the procedures were performed according to the relevant laws and guidelines. Informed consent was obtained from all human research participants. All ethical regulations relevant to human research participants were followed. The animal experiments were approved by the Laboratory Animal Welfare and Ethical Review of China medical University (No. CMU2020186). We have complied with all relevant ethical regulations for animal use.

### Recruitment of participants and sample collection

We recruited 23 initially untreated patients with HT and 25 healthy individuals from the First Affiliated Hospital of China Medical University. The two groups were matched for age and sex. Blood samples were collected from the participants in the morning. Serum thyroid-stimulating hormone (TSH), thyroid peroxidase antibody (TPOAb), and thyroglobulin antibody (TgAb) levels were measured using an electrochemiluminescence immunoassay on a Cobas 601 analyzer (Roche Diagnostics, Basel, Switzerland) at the Central Laboratory in Shenyang. An endocrinologist diagnosed and verified all patients with HT based on physical examination and biochemistry. The inclusion criteria for patients with HT were as follows: age 18–65 years and the presence of euthyroidism (normal FT3, FT4, and TSH plasma levels, without hormone therapy). The normal ranges for FT3, FT4, TSH, TPOAb, and TgAb provided by the manufacturers were 2.43–6.01 pmol/L, 9.01–19.05 pmol/L, 0.35–4.94 mIU/L, <5.61 IU/mL, and <4.11 IU/mL, respectively. The exclusion criteria were as follows: age <18 years; use of iodine drugs within the previous 3 months; pregnancy; use of antibiotics; probiotics; or prebiotics within the previous 3 months; use of hormonal medication or Chinese herbal medicine; chronic diarrhea; and history of inflammatory disease (Table 1). The patients collected the fecal samples using disposable sterile forceps in the morning and stored them at −80 °C after liquid nitrogen freezing until DNA extraction.

**Table 1:** Clinical characteristics of patients with HT and healthy control group.

	Control	HT	*P* value
Number	25	23	-
Sex (M/F)	13/12	10/13	ns
Age (Y)	43.83 ± 12.26	42.70 ± 11.20	ns
TPOAb (IU/ml)	1.15 ± 1.18	1580.42 ± 1462.93	^b^
TgAb (IU/ml)	1.41 ± 0.92	271.10 ± 267.80	^b^
TSH (mIU/L)	1.99 ± 0.93	2.85 ± 1.20	^a^
FT3 (pmol/L)	4.70 ± 0.40	4.33 ± 0.45	^a^
FT4 (pmol/L)	12.93 ± 1.07	13.01 ± 1.68	ns

Data are shown as the mean ± SD (Standard Deviation). The data normality was tested using the Shapiro-Wilk normality test. If data was not normally distributed, the Mann−Whitney *U* test was used. The t-test (FT3, FT4) and Mann-Whitney U test (Age, TPOAb, TgAb, TSH) were used to detect the clinical characteristics of patients with HT and healthy individuals.

^a^*P* < 0.01.

^b^*P* < 0.0001, HT, Hashimoto thyroiditis; M, male; F, Female; Y, year; ns, no significance.

### HT mouse model introduction and evaluation

Hematoxylin-Eosin (HE) staining was performed to measure thyroid gland aberrations. The HT mice exhibited thyroid lymphocyte infiltration and follicle expansion and lesions. The thyroid glands were collected and fixed in 4% paraformaldehyde and embedded into paraffin, which was sliced into 5-μm-thick sections and stained with HE staining. The extent of lymphocyte infiltration in the thyroid sections was scored with a scale from 0 to 5 as previously described^43^: 0, no infiltration; 1,1–10% infiltration; 2,10–30% infiltration; 3, 30% −50% infiltration; 4, extensive infiltration greater than 50% of total area. The scores were determined from at least 3 nonconsecutive thyroid tissue slices.

### ELISA analysis of TgAb and LPS

The level of serum mTg-specific IgG antibody was detected by enzyme-linked immunosorbent assay (ELISA) as previously reported^43,44^. The serum from each mouse was diluted 1:200 with PBS. Peroxidase-labeled goat anti-mouse IgG diluted 1:1000 (SA00001-1, Proteintech) was added. The microplate reader (Bio-Rad 680, US) was used to read optical density values at 450 nm. Lipopolysaccharide (LPS), the gram-negative bacterial endotoxin, was detected using the ELISA kit purchased from Jianglai Biotechnology (JL20691), Shanghai, China. The experimental procedures were caried out according to the manufacturer’s instruction. The concentrations of target proteins were determined by standard protein curves.

### Animals and experimental design

NOD.H-2h4, the spontaneously develop thyroiditis mouse model was purchased from Jackson Laboratory Inc, USA. Acclimatized (12-h light/dark cycle) under standard conditions (temperature 22 ± 2 °C, humidity 50%–60%) in the Specific pathogen Free laboratory of China medical university. All experiments were conducted following the principles and procedures outlined for the care and use of laboratory animals. In this study, male and female NOD.H-2h4 mice aged 5–6 weeks were used. To explore the influence of iodine intake on the composition of the gut microbiota of HT mice, mice were given 5 mg/L, 50 mg/L, and 500 mg/L sodium iodide (NaI) in their drinking water for 10 weeks, and the control group received regular drinking water throughout the experiment. HE staining of the thyroid and serum TgAb concentrations were used to prove the HT model. Fecal samples from the constructed HT model with different iodine intakes were collected to investigate the microbiota composition using 16S rRNA sequencing. Overall, 51 mice were used to explore the influence of iodine intake on the composition of the gut microbiota as follows: the control (*n* = 12), the 5 mg/L (*n* = 10), the 50 mg/L (*n* = 10), and 500 mg/L (*n* = 10) groups. During the experiments, sodium butyrate (120 mM, Sigma, 303410) was added to the butyrate group (*n* = 9). We used gas chromatography-mass spectrometry (GC-MS) to explore the differences in SCFA between the HT (*n* = 8) and the control (*n* = 8) groups.

### Untargeted metabolomic analysis using LC-MS

Plasma samples (100 μL) and prechilled methanol (400 μL) were mixed by well vortexing. A some of supernatant was diluted to final concentration containing 53% methanol by liquid chromatograph-mass spectrometer (LC-MS) grade water. The samples were subsequently transferred to a fresh Eppendorf tube and then were centrifuged at 15,000 g, 4 °C for 10 min. Finally, the supernatant was injected into the LC-MS/MS system analysis. LC-MS/MS analyses were performed using a Vanquish UHPLC system (Thermo Fisher) coupled with an Orbitrap Q Exactive series mass spectrometer (Thermo Fisher). Samples were injected onto an Hyperil Gold column (100 × 2.1 mm, 1.9 μm) using a 16- min linear gradient at a flow rate of 0.2 mL/min. The eluents for the positive polarity mode were eluent A (0.1% FA in Water) and eluent B (Methanol). The eluents for the negative polarity mode were eluent A (5 mM ammonium acetate, pH 9.0) and eluent B (Methanol). The solvent gradient was set as follows: 2% B, 1.5 min; 2–100% B, 12.0 min; 100% B, 14.0min; 100−2% B, 14.1min; 2% B, 17 min. The raw data files generated by UHPLC-MS/MS were processed using the Compound Discoverer 3.1 (CD3.1, Thermo Fisher) to perform peak alignment, peak picking, and quantitation for each metabolite. The main parameters were set as follows: retention The KEGG database, HMDB and LIPIDMaps database were used to annotate the identified metabolites. For multivariate statistical analysis, MetaX was used to transform the data and then perform the partial least squares discriminant analysis (PLS-DA). The fold change (FC value) of the levels of metabolites between the two groups was calculated. Metabolites with VIP > 1, *P* value < 0.05 and fold change (FC) ≥ 2 or FC ≤ 0.5 were considered to be differential metabolites.

### 16SrRNA gene sequencing

Total genome DNA from samples was extracted using CTAB method. DNA concentration and purity was monitored on 1% agarose gels. According to the concentration, DNA was diluted to 1 ng/µL using sterile water. Fecal DNA samples from each group were selected randomly for 16S rRNA gene sequencing and analysis. The V3-V4 regions was amplified with the primers 341 F (5’-CCTAYGGGRBGCASCAG-3’), and 806 R (5’-GGACTACNNGGGTATCTAAT-3’), and sequencing by the IIIumina NovaSeq platform (IIlumina, San Diego, CA, USA). All PCR reactions were carried out with 15 µL ofPhusion® High -Fidelity PCR Master Mix (New England Biolabs); 2 µM of forward and reverse primers, and about 10 ng template DNA. Thermal cycling consisted of initial denaturation at 98 °C for 1 min, followed by 30 cycles of denaturation at 98 °C for 10 s, annealing at 50 °C for 30 s, and elongation at 72 °C for 30 s. Sequences analysis were performed by Uparse software (Uparse v7.0. 1001). Sequences with ≥97% similarity were assigned to the same Operational taxonomic Unit (OTU). Representative sequence for each OTU was screened for further annotation., then QIIME software (V1.9.1) was used for OTU clustering and species annotation, α-diversity, β-diversity, and diversity index analysis were performed based on the results of OTU cluster analysis. The predominant bacterial community difference between groups was detected using the LDA effect size.

### Targeted metabolomic analysis of SCFAs using GC-MS

Blood samples collected from HT patients and healthy individuals were centrifuged at 1500 g for 10 min 4 °C to collect serum. Samples were extracted in 50 μL of 15% phosphoric acid with 10 μL of 75 μg/mL 4-methylvaleric acid solution as IS and 14 0 μL ether. Subsequently, the samples were centrifuged at 4 °C for 10 min at 12,000 rpm after vortexing for 1 min and the supernatant was transferred into the vial prior to GC-MS analysis. Fecal sample collected from mice were prepared by dissolving fecal samples in ultrapure water and mixed on a vortex mixer, and then centrifuged at 4800 rpm for 20 min at 4 °C. The supernatant was collected and analyzed by GC analysis. GC analysis was performed on an Agilent 7890 A GC system equipped with a flame ionization detector (FID) (Agilent Technologies Inc., USA). A GC column was used for measuring SCFAs. The initial oven temperature was 80 °C for 0.5 min, and then was raised to 150 °C by 4 °C /min, subsequently raised to 230 °C at a rate of 20 °C/min, and finally maintained at 230 °C for 10 min. As the carrier gas, nitrogen was applied at a flow rate of 19.0 mL/min. The temperatures of the FID and injection port were 240 °C. The flow rates of hydrogen and synthetic air were 30 and 300 mL/min, respectively. The injected sample volume for GC analysis was 0.3 μL, and the running time for each analysis was 32 min. The results were analyzed with an Agilent ChemStation plus software.

### Quantitative real-time PCR

Total RNA was extracted using the TRIzol reagent (TaKaRa, Kusatsu, Japan) and revers transcribed into mRNA by PrimeScript RT reagent Kit 036 A (TaKaRa, Kusatsu, Japan), and quantified by SYBR Premix Ex TaqTM 820 A (TaKaRa, Kusatsu, Japan). Data normalization of each sample was performed according to the GAPDH level. RT-qPCR analysis was carried out with a LightCycler 480 system. The primer sequences are listed as follows: *FFAR3*(F: TCCTGGCATCGGCTCACTGTAG; R: ACGGACTCTCACGGCTGACATAG), and *FFAR2*(F: GCTGACAGGCTTCGGCTTCTAC; R: CTGCTCTTGGGTGAAGTTCTCGTAG).

### Flow cytometric analysis

For Th1/Th2/Th17/Treg cell staining, spleen mononuclear cells were obtained and incubated with cell activation cocktail (00-4975-03, Invitrogen)at 37 °C for 5 h. Then the cells were incubated with Fc-block at room temperature for 10 min to avoid non-specific binding. To detect extracellular proteins, anti-mouse CD4-FITC (11-0041-82, 0.25 μg/test, Invitrogen) and anti-mouse CD25-APC (17-0251-81, 0.125 μg/test, Invitrogen). The True-Nuclear Transcription Factor Buffer Set (00-5523-00, Invitrogen) was applied before anti-mouse Foxp3-PE (12-5773-82, 1 μg/test, Invitrogen). For Th1/Th2/Th17 cell assay, PBMCs were stained with intracellular IFN-γ-APC (17-0251-81, 0.125 μg/test, Invitrogen), IL-4-PE (12-7041-82, 0.125 μg/test, Invitrogen), and IL-17- PerCP (45-7177-82, 0.03 μg/test, Invitrogen).The lymphocyte cells were tested with FACScan Flow Cytometry and WinMDI12.9 and analyzed by FlowJo X software. Each sample was set to analyze 50,000 cells. The gating strategy is provided in the Supplementary Fig. S8.

### Mice colon Alcian blue/periodic acid-Schiff staining and HE staining

For each mouse, three sections were selected to be stained with Alcian Blue Periodic acid Schiff (AB/PAS) and HE staining. AB/PAS staining was performed using the panel (Solarbio, G1285). The prepared sections were treated with Alcian blue dye for 10 min following deparaffinization and rehydration, then washed with running water for 5 min, and subsequently stained with periodic acid for 3 min. The sections were oxidized and washed twice, followed by staining with Schiff’s reagent for 12 min. After a 10-min rinse in distilled water, the sections underwent hematoxylin staining. They were then treated with Scott’s Bluing Solution for 3 min and washed for an additional 3 min. Finally, the sections were dehydrated using a series of ethanol washes and cleared with xylene before sealing. The goblet cell was observed using AB/PAS staining.

The colon tissue was fixed in 10% neutral buffered formalin for 24 h, subsequently dehydrated, and then embedded in paraffin wax. Approximately 5 μm sections of the paraffin-embedded tissue were cut and stained with HE (Solarbio, G1120). The goblet cells are round cells that appear clear on HE staining. All the sections were observed and photographed under a light microscope (Olympus, Tokyo, Japan).

### Bioinformatic analysis

Total genome DNA from samples was extracted using CTAB method. Quality filtering on the raw tags were performed under specific filtering conditions to obtain the high-quality clean tags according to the QIIME (V1.9.1). Sequence analyses were performed using Uparse software (Uparse v7.0. 1). Sequences with ≥97% similarity were assigned to the same OTUs. Representative sequence for each OTU was screened for further annotation. All the а diversity indices in our samples were calculated with QIIME (Version 1.7.0) and displayed with R software (Version 2. 15.3). Distance matrices (beta diversity) between samples were assessed using principal coordinate analysis (PCoA), which was displayed by ade4 package and ggplot2 package in R software (Version 2. 15.3). The linear discriminant analysis effect size (LEfSe) analysis was performed to identify differentially abundant microbiota (Linear discriminant analysis (LDA) score >3, *P* < 0.05). Random Forest was applied to distinguished the genus-level abundance of microbiota between HT patients and healthy controls.

### Statistics and reproducibility

Data are presented as mean ± standard deviation. All experiments were repeated at least three times. Statistical analysis for multiple comparisons was performed using a one-way analysis of variance. The nonparametric Kruskal-Wallis test was used when the data did not fit the normal distribution. An independent sample *t* test was used when the data fit the normal distribution for a two-group comparison and Mann−Whitney U test was used if data did not fit normal distribution. The analysis was performed using GraphPad Prism version 9.4.0 for windows (GraphPad, San Diego, USA). Pearson’s correlation analysis or Spearman’s correlation analysis was conducted to determine the correlations between different experiments. ^*^P < 0.05, ^**^P < 0.01, ^***^P < 0.001, and ^****^P < 0.0001, ns, no significance.

### Reporting summary

Further information on research design is available in the Nature Portfolio Reporting Summary linked to this article.

### Supplementary information

Supplementary information

Description of Additional Supplementary Files

Supplementary Data 1

Supplementary Data 2

Reporting summary

## Data Availability

All the 16S sequencing data generated in our study are publicly available on the Sequence Read Archive (SRA) of the National Center for Information (NCBI) with the accession number PRJNA1033804. The metabolism data in this study was deposited in Metabolights with the accession number MTBLS8813, which is an open-access database repository for metabolomics data. Source data related to plots and graphs in the manuscript can be found in Supplementary Data 1. Source data of clinical characteristics of patients with HT and healthy control groups used in Table 1 is available in Supplementary Data file 2. Any raw data will be available from the corresponding author upon reasonable request.
