# Supplementary material for: Effects of iodine intake on gut microbiota and gut metabolites in Hashimoto thyroiditis-diseased humans and mice
Source: Commun Biol. 2024 Jan 29;7:136. doi: 10.1038/s42003-024-05813-6 (PMC10824742; doi:10.1038/s42003-024-05813-6)
Supplement: Supplementary file 1 — Supplementary information [file 42003_2024_5813_MOESM1_ESM.pdf]

Supplementary information for

Effects of iodine intake on gut microbiota and gut metabolites in Hashimoto thyroiditis-diseased humans and mice

Authors:

Boshen Gong[1#], Fanrui Meng[1#], Xichang Wang[1], Yutong Han[1], Wanyu Yang[1], Chuyuan Wang [1*] Zhongyan Shan [1*]

Affiliations:

[1]Department of Endocrinology and Metabolism, The First Affiliated Hospital of China Medical University, China Medical University, Shenyang, China.

[#]These authors contributed equally.

[*]Corresponding author:

Zhongyan Shan

Email: shanzhongyan@medmail.com.cn

Chuyuan Wang

Email: Chuyuan0215@163.com

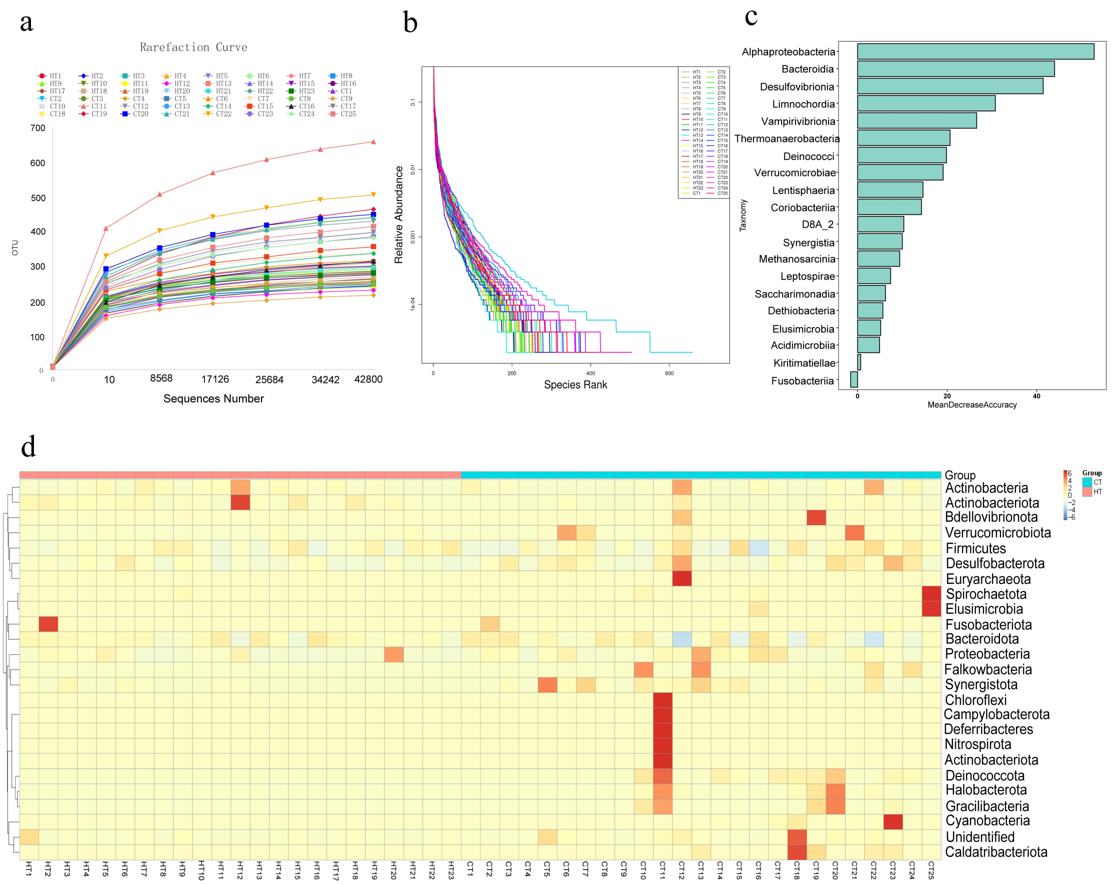

Fig S1. a) Rarefaction curves of the gut microbiota at the OTU level. **b)** Rank-abundance curves of the gut microbiota at the OTU level. **c)** Random Forests analysis of gut microbiota. The genus level of bacteria could significantly discriminate between HT patients and healthy control were presented in descending order. **d)** Heatmap of the relative abundances of microbiota at the phylum level in HT and control groups. The color bar indicates the Z score, which represents the relative abundance. Z score < (> 0) illustrates that the relative abundance was lower (higher) than the mean.

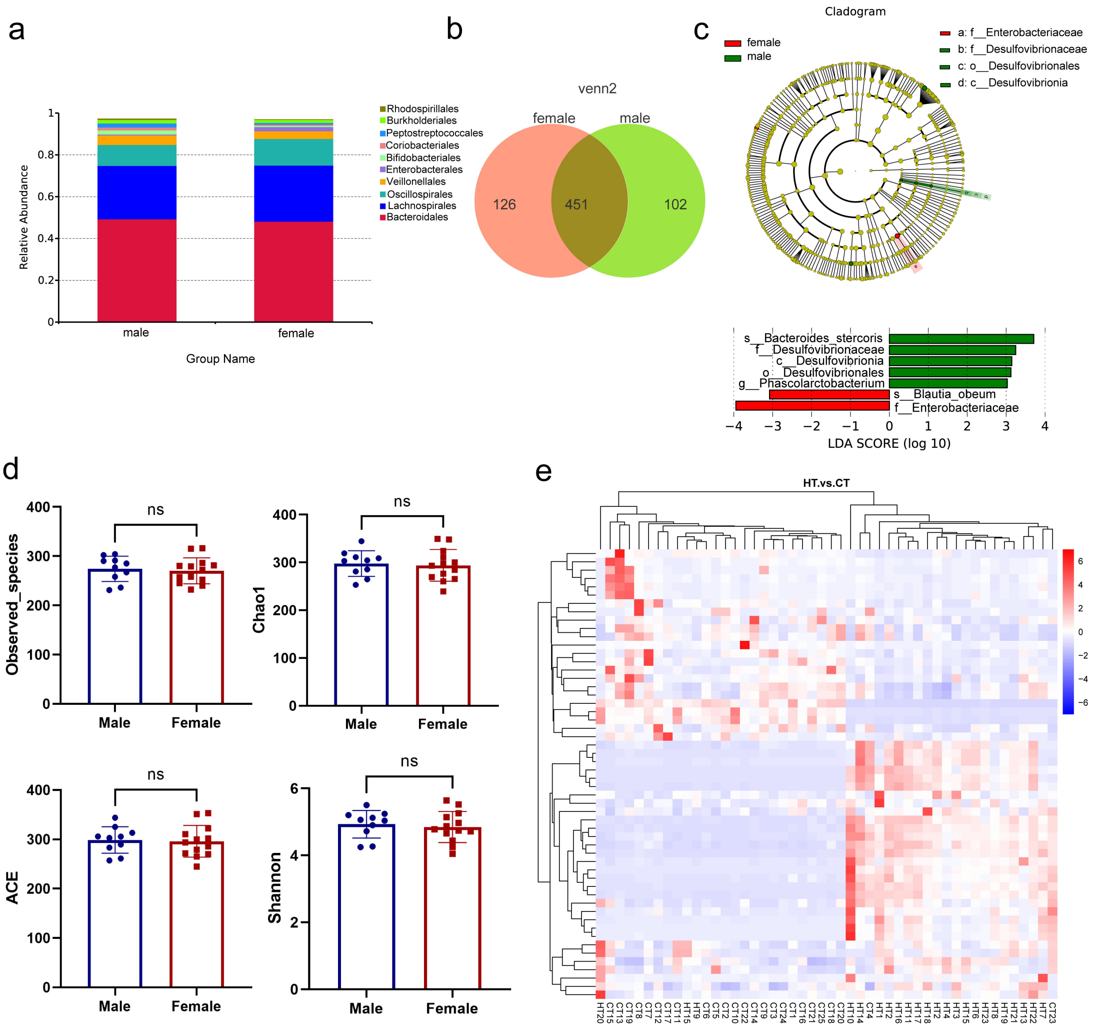

Fig S2. a) Order level comparison of fecal microbiota between male and female groups with HT. (N=10 for male group, and 13 for female group). **b)** The Venn diagram illustrates the overlap of OTUs in gut microbiota between different sex groups with HT. **c)** Cladogram generated using LEfSe analysis. LDA scores of the microbiota between two sex groups at different taxonomic levels using LEfSe analysis. LDA score >3 or <3 represents bacteria taxa that are significantly enriched (P < 0.05). **d)** The α diversity indices of the microbiota between male and female groups with HT. **e)** Heatmap of the differentially expressed metabolites, which

significantly changed in the HT group. The color bar indicates the Z score, which represents the relative abundance. Z score <0 (>0) meant the relative abundance was lower (higher) than the mean.

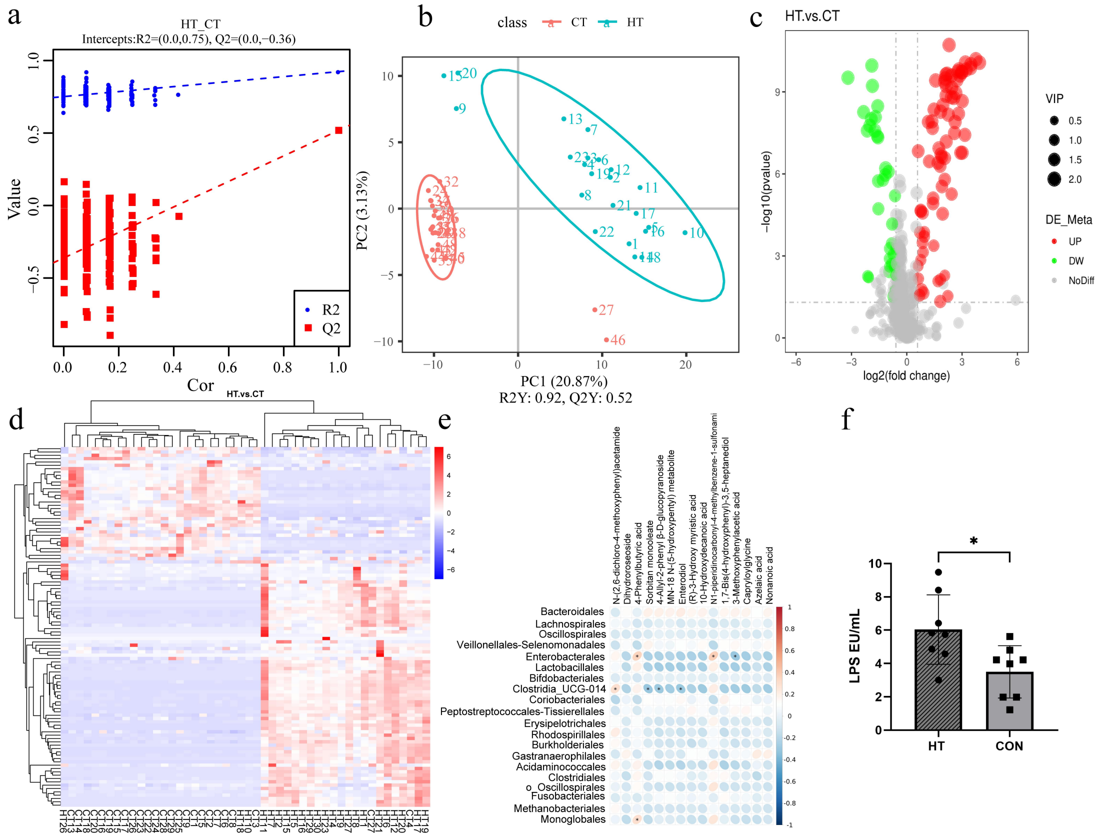

Fig S3. a) The permutation test between the control and HT groups (positive mode). **b)** PLS-DA between the control and HT groups (positive mode). **c)**Volcano map of the metabolites screened between the HT and control groups of positive mode. **d)** Heatmap of the differentially expressed metabolites which significantly changed in HT group of positive mode (corrected $P < 0.05$ by t-test). The color bar indicates Z score, which represents the relative abundance. Z score <0 (>0) meant the relative abundance was lower (higher) than the mean. **e)** Heatmap of correlations between the significantly changed gut microbiota and 15 metabolites in patients with HT. The color bar with numbers indicates the correlation coefficients. **f)** Serum levels of LPS

in mice with HT and healthy control group. The t-test was used to detect significant differences between two groups. $^{*}P<0.05$. N=8 in each group.

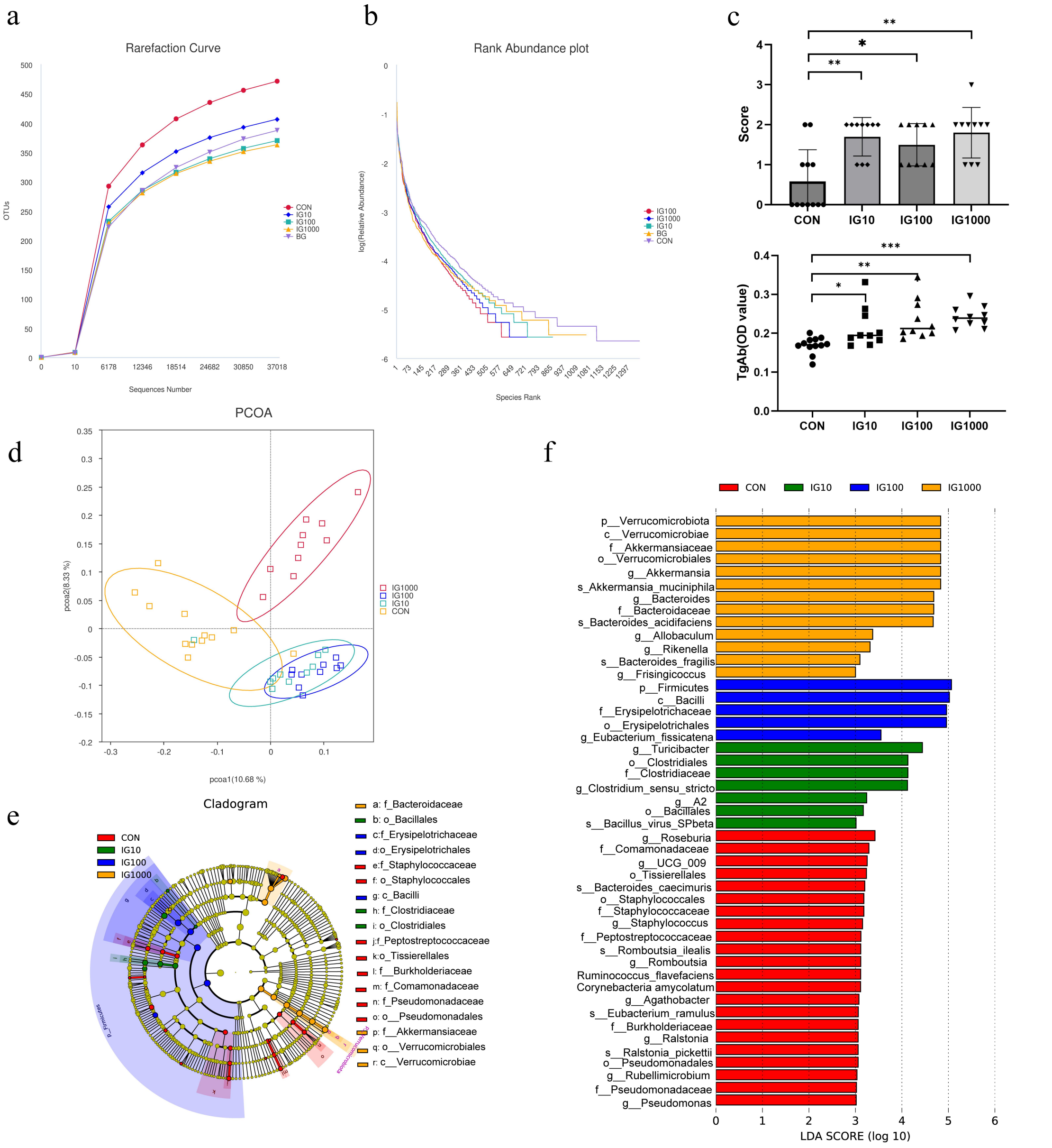

Fig S4. a) Rarefaction curves of the gut microbiota at the OTU level. **b)** Rank-abundance curves of the gut microbiota at the OTU level. **c)** The scoring of lymphocyte infiltration of thyroid gland serum. Serum TgAb concentrations were determined using ELISA. The Kruskal-Wallis test was used to detect significant differences among four groups. $^{*}P<0.05$, $^{**}P<0.01$, $^{***}P<0.001$. **d)** PCoA plot based on

binary_jaccard distance showing microbiota diversity among these four groups. **e)** Cladogram generated using LEfSe analysis. Red: increased abundance in the control group. Green: increased abundance in the IG10 group. Blue: increased abundance in the IG100 group. Yellow: increased abundance in the IG1000 group. **f)** LDA scores of the microbiota among these four groups at different taxonomic levels using LEfSe analysis. LDA score >3 or <3 represents bacteria taxa that are significantly enriched in the HT group (green) or control group (red) (P < 0.05). N=12 for CON group, 10 for IG10 group, 10 for IG100 group, and 10 for IG1000 group.

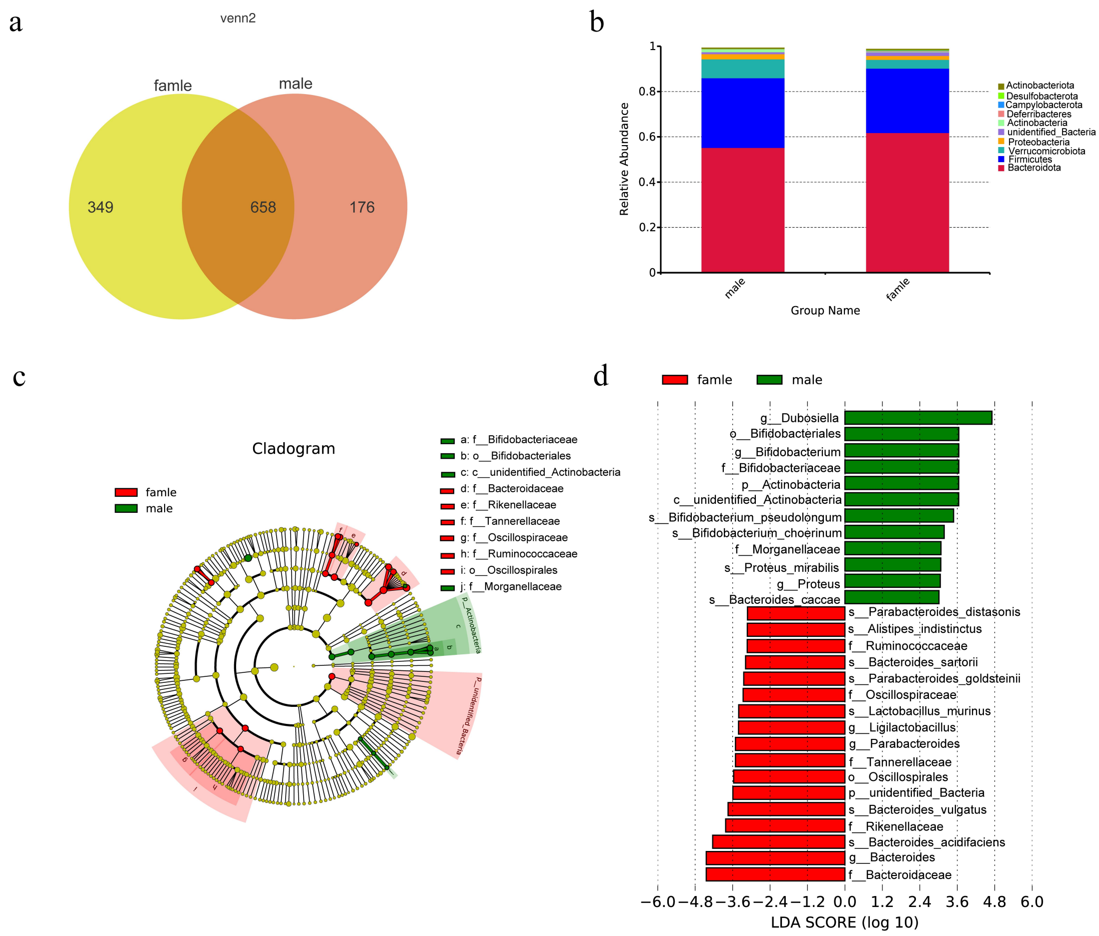

Fig S5. a) The Venn diagram illustrates the overlap of OTUs in gut microbiota between different sex groups of mice with HT. **b)** Phylum level comparison of fecal microbiota between male and female mice groups with HT. **c)** LDA scores of the

microbiota in male and female groups with HT at different taxonomic levels using LEfSe analysis. LDA score >3 or <3 represents bacteria taxa that are significantly enriched in the male group (green) or female group (red) ($P < 0.05$). **d)** Cladogram generated using LEfSe analysis. Red: increased abundance in the female group. Green: increased abundance in the male group.

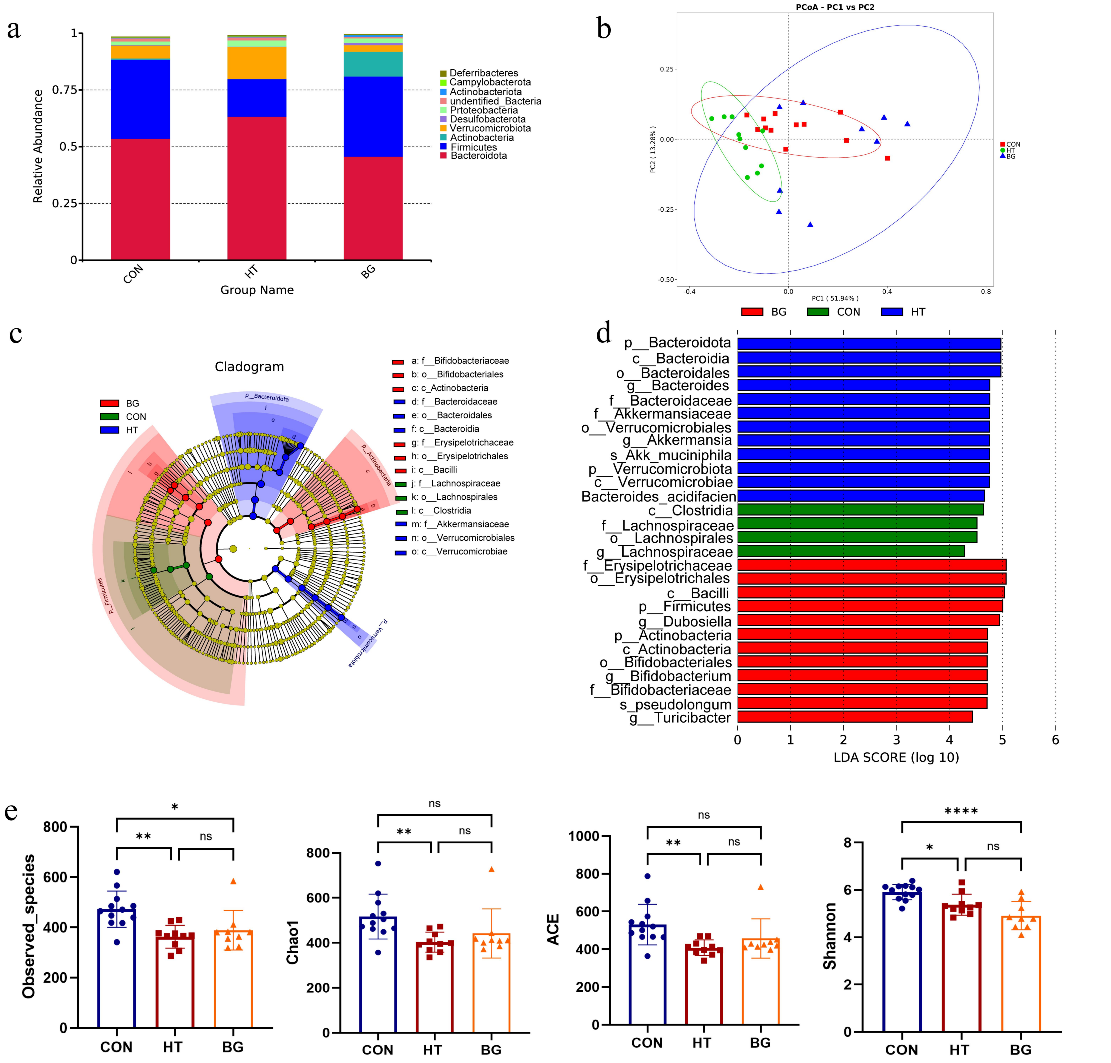

Fig S6. a) Phylum level comparison of relative abundance fecal microbiota among healthy, HT, and butyrate groups. **b)** PCoA plot based on binary_jaccard distance showing microbiota diversity among these three groups. **c)** Cladogram generated using LEfSe analysis. Red: increased abundance in the BG group. Green: increased

abundance in the CON group. Blue: increased abundance in the HT group. **d)** Cladogram generated using LEfSe analysis. LDA scores of the microbiota among three groups at different taxonomic levels using LEfSe analysis. LDA score >3 or <3 represents bacteria taxa that are significantly enriched ($P < 0.05$). **e)** The α diversity indices (Chao 1, ACE, Shannon, and Simpson indexes) of the microbiota among these three groups. N=12, 10, 9 for CON, HT, and BG groups respectively.

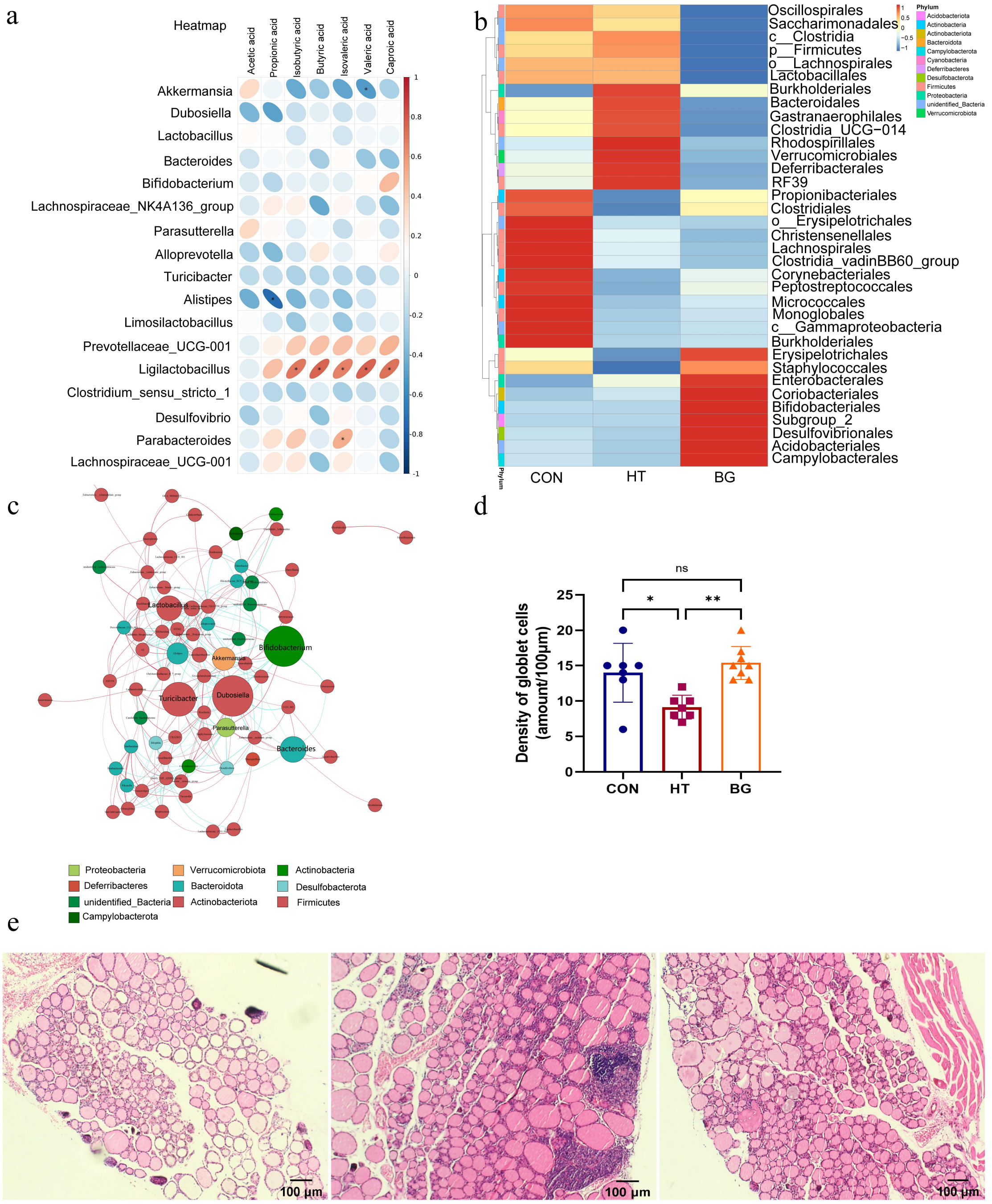

Fig S7.a) Heatmap of correlations between the significantly changed gut microbiota and 7 SCFA metabolites in HT mice at genus level. The color bar with numbers indicates the correlation coefficients. **b)** Heatmap based on the abundance of the first 35 order among these three groups. **c)** Network of the relative abundance of butyrate group at genus level. The size of the node represents the relative abundance of microbiota, the color of the node represents different genus. **d)** Histogram presenting the statistical result of goblet cell density. The data are expressed as the mean ± standard deviation. One-way ANOVA was used to detect significant differences. $^{*}P < 0.05$, $^{**}P < 0.01$. N=7, 7, 8 for CON, HT, and BG groups respectively. **e)** Thyroid inflammation was determined according to the lymphocyte infiltration area using HE staining of the mice's thyroid gland. Scale bar: 100 μm.

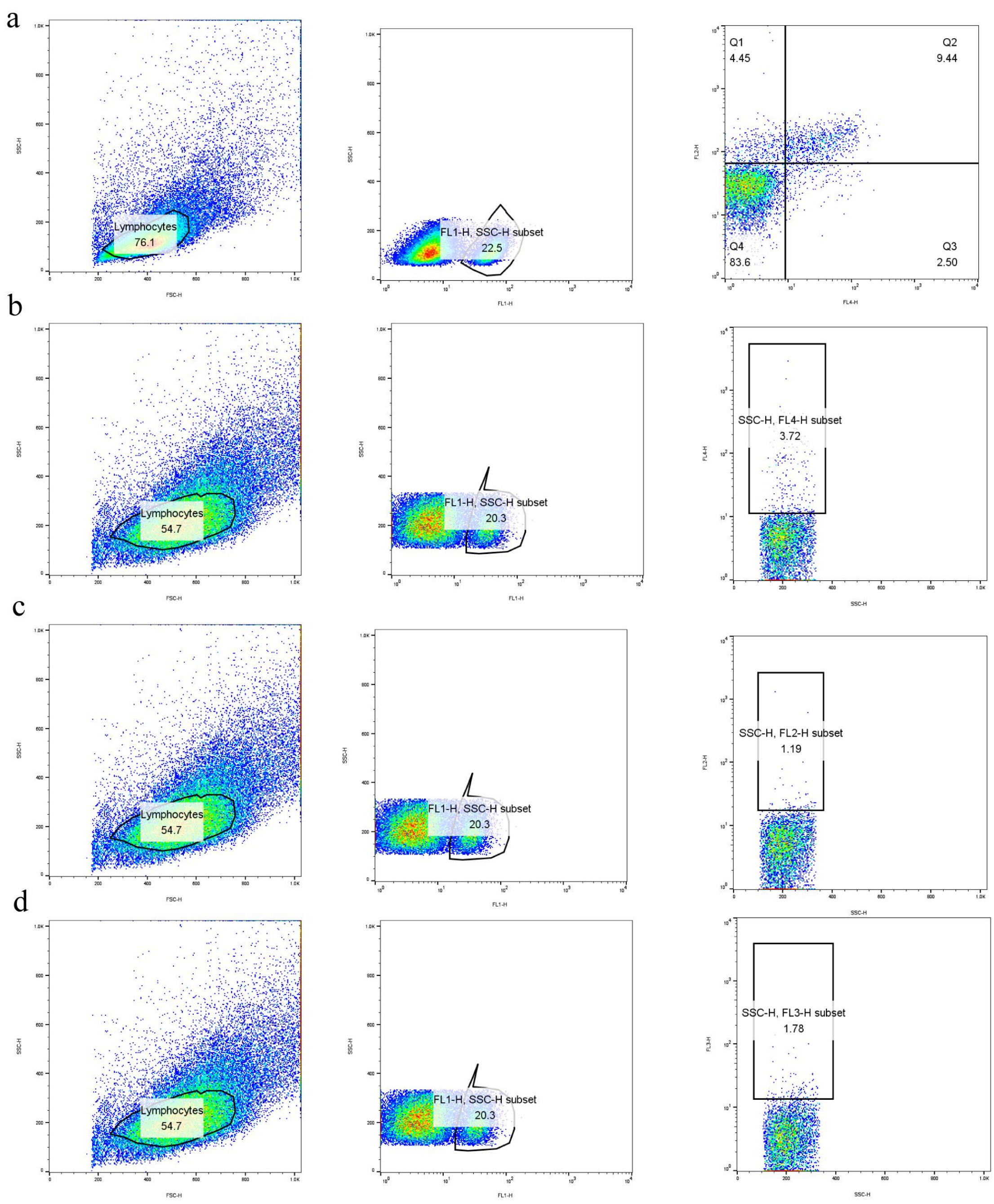

Fig S8. The flow cytometer gating strategy for **a)** CD4+CD25+FOXP3+ cells (Treg), **b)** CD4+ IFN^{+} cells (Th1), **c)** CD4+ IL4+ T cells (Th2), **d)** CD4+ IL17+ cells (Th17).
