# Supplementary material for: Effects of iodine intake on gut microbiota and gut metabolites in Hashimoto thyroiditis-diseased humans and mice
Source: Commun Biol. 2024 Jan 29;7:136. doi: 10.1038/s42003-024-05813-6 (PMC10824742; doi:10.1038/s42003-024-05813-6)
Supplement: Supplementary file 2 — Description of Additional Supplementary Files [file 42003_2024_5813_MOESM2_ESM.pdf]

## Description of Additional Supplementary Files

**File name:** Supplementary Data 1
**Description:** Targeted metabolomic analysis of SCFAs and microbiota data used in the figures.

**File name:** Supplementary Data 2
**Description:** Source data of clinical characteristics of patients with HT and healthy control groups used in the study.

**File name:** Supplementary Data 3
**Description:** The source data for Fig. 6a-h, Fig. S3f, Fig. S4c, Fig. S6e, Fig. S7d.
